# Mouse model of Angelman syndrome exhibits reduced retinal activity during development

**DOI:** 10.3389/fnins.2026.1895084

**Published:** 2026-07-15

**Authors:** Rishikesh Kumar Gupta, Alexander J. Fisch, Fernanda S. Orsi, Alexandre Tiriac

**Affiliations:** 1 Department of Biological Sciences, Vanderbilt University, Nashville, TN, United States; 2 Vanderbilt Brain Institute, Vanderbilt University, Nashville, TN, United States; 3 Department of Pharmacology, Vanderbilt University, Nashville, TN, United States; 4 Department of Ophthalmology and Visual Sciences, Vanderbilt University Medical Center, Nashville, TN, United States

**Keywords:** Angelman syndrome (AS), E3 ligase, Microlectrode array, neurodevelopment, retinal waves

## Abstract

Angelman Syndrome (AS) is a neurodevelopmental disorder characterized by intellectual disability, seizures, and motor dysfunction caused by loss of function of the maternal UBE3A allele. A large proportion of patients exhibit visual impairments, but the underlying circuit-level mechanisms are not well understood. Spontaneous retinal waves provide essential patterned activity that guides the wiring and refinement of retinofugal projections to visual brain centers during a critical early developmental window. Here, using high-density microelectrode array recordings from postnatal day 8–11 mouse retinas, we demonstrate that *Ube3a* deficiency leads to reduced retinal ganglion cell (RGC) firing rates and fewer retinal waves while largely preserving retinal wave propagation properties. The reduced activity levels observed in AS retinas represent a novel finding that may help explain how *Ube3a* deficiency disrupts the normal activity-dependent refinement of developing neural circuits.

## Introduction

Angelman syndrome (AS) is a neurodevelopmental disorder, affecting ~1 in 15,000 individuals (Margolis et al., 2015). The primary genetic cause of AS is the loss of function of the maternal allele of the *Ube3a* gene (Williams et al., 1990; Wallace et al., 2012), where the maternal allele is expressed in neurons (Vihma et al., 2024) and the paternal allele is silenced by imprinting (Jiang et al., 2010). The *Ube3a* gene, located on chromosome 15q11-q13, encodes UBE3A, an E3 ubiquitin ligase that tags substrate proteins for proteasomal degradation (Greer et al., 2010). Through this function, UBE3A regulates synaptic development, function, and plasticity during critical periods of brain development (Jiang et al., 1998; Ozarkar et al., 2024). The core clinical features of AS include epilepsy, motor dysfunction, intellectual disability (Jiang et al., 1998), and speech impairment (Pelc et al., 2008). Notably, AS is also associated with a high prevalence of congenital visual deficits, including visual impairment such as nystagmus, strabismus, and significant refractive errors like high levels of hyperopia and astigmatism (Michieletto et al., 2011; Galli et al., 2023). These abnormalities in visual behaviors indicate a potential deficit in the initial establishment or refinement of the retina-to-brain circuitry, a process heavily dependent on spontaneous retinal activity (Huberman et al., 2008; Thompson et al., 2017). Therefore, a mechanistic understanding of how retinal activity during development is impacted in AS is needed.

The development of precise neural connectivity in the mammalian brain is not solely genetically predetermined but is influenced by spontaneous, correlated neural activity. In the developing visual system (approximately postnatal days 0–14 (P0–P14) in mice), this process is initiated in the eye, prior to sensory experience, through highly patterned, spontaneous bursts of action potentials that sweep across the retina, known as retinal waves (Feller, 1999; Ackman et al., 2012). Because retinal circuitry matures progressively as neurons across successive layers establish synaptic connections, the spatiotemporal properties of retinal waves change throughout postnatal development (Maccione et al., 2014; Ge et al., 2021; Yeager et al., 2025). Retinal waves progress through three distinct developmental stages in mice, each defined by different neurotransmitter systems and cellular mechanisms (Feller, 1999; Blankenship and Feller, 2010). Stage I waves (E17-P2 in mice) are mediated by gap junctions and cholinergic circuits (Feller, 1999; Voufo et al., 2023). Stage II waves (~P0–P9) are initiated and propagated primarily by cholinergic transmission from starburst amacrine cells (SACs), which provide recurrent excitatory input to neighboring SACs and RGCs (Feller et al., 1996; Zheng et al., 2006; Ford et al., 2012). Stage III waves (~P10–P14) are mediated by glutamatergic transmission from bipolar cells as conventional synaptic circuitry matures and the retina transitions to light-responsive circuitry (Blankenship and Feller, 2010; Tiriac et al., 2018; Ge et al., 2021). Stages II and III overlap during the second postnatal week (P8–P12), providing a unique window in which both wave-generating mechanisms are simultaneously active (Maccione et al., 2014). Since UBE3A regulates synaptic protein turnover and neuronal excitability across multiple cell types (Greer et al., 2010), examining retinal activity during this overlapping period, serves as an ideal starting point to determine whether *Ube3a* deficiency differentially impacts fundamental mechanisms of network activity, regardless of whether the primary vulnerability lies in cholinergic, glutamatergic, or shared mechanisms.

Importantly, the dynamic spatiotemporal properties of retinal waves provide instructive signals that guide the activity-dependent wiring to establish eye-specific segregation (Stellwagen and Shatz, 2002), retinotopic maps (Chandrasekaran et al., 2005; Matsumoto et al., 2024), and the emergence of direction-selective circuits (Stafford et al., 2009; Ge et al., 2021). Consequently, perturbations in retinal wave patterning can lead to errors in topographic mapping (McLaughlin et al., 2003), resulting in functional visual deficits (Burbridge et al., 2024). Beyond shaping neuronal connectivity, retinal waves also influence non-neuronal processes, including vascular development (Weiner et al., 2019; Biswas et al., 2024). Although disruptions in retinal wave dynamics have been implicated in neurodevelopmental disorders, the mechanisms underlying these visual abnormalities remain unclear, including whether the spatiotemporal properties of retinal waves are altered in AS.

The murine model of AS, which carries a maternal deletion of *Ube3a* (*Ube3a^m−/p+^*), recapitulates many core features of the human disorder, including deficits in visual brain areas (Yashiro et al., 2009; Wallace et al., 2012; Townsend et al., 2020), and has been instrumental in elucidating underlying neural mechanisms (Shi et al., 2015; Sidorov et al., 2018; Ozarkar et al., 2024; Vihma et al., 2024). Given the established role of *Ube3a* in regulating synaptic function and network activity, we asked whether deletion of the maternal Ube3a allele disrupts retinal wave activity and/or alters wave spatiotemporal properties. To test this, we performed high-density microelectrode array (HD-MEA) recordings on isolated retinas from a well-characterized mouse model of AS during P8-P11. We conducted a comprehensive analysis of both single-neuron firing properties and the network-level dynamics of thousands of individual wave events. We found that overall activity levels, including single-neuron firing rates and wave frequency, were reduced in AS mice but that the spatiotemporal properties of the remaining retinal waves were mostly intact.

## Results

### AS mice exhibit reduced RGC firing rates and fewer retinal waves

**Figure 1:**
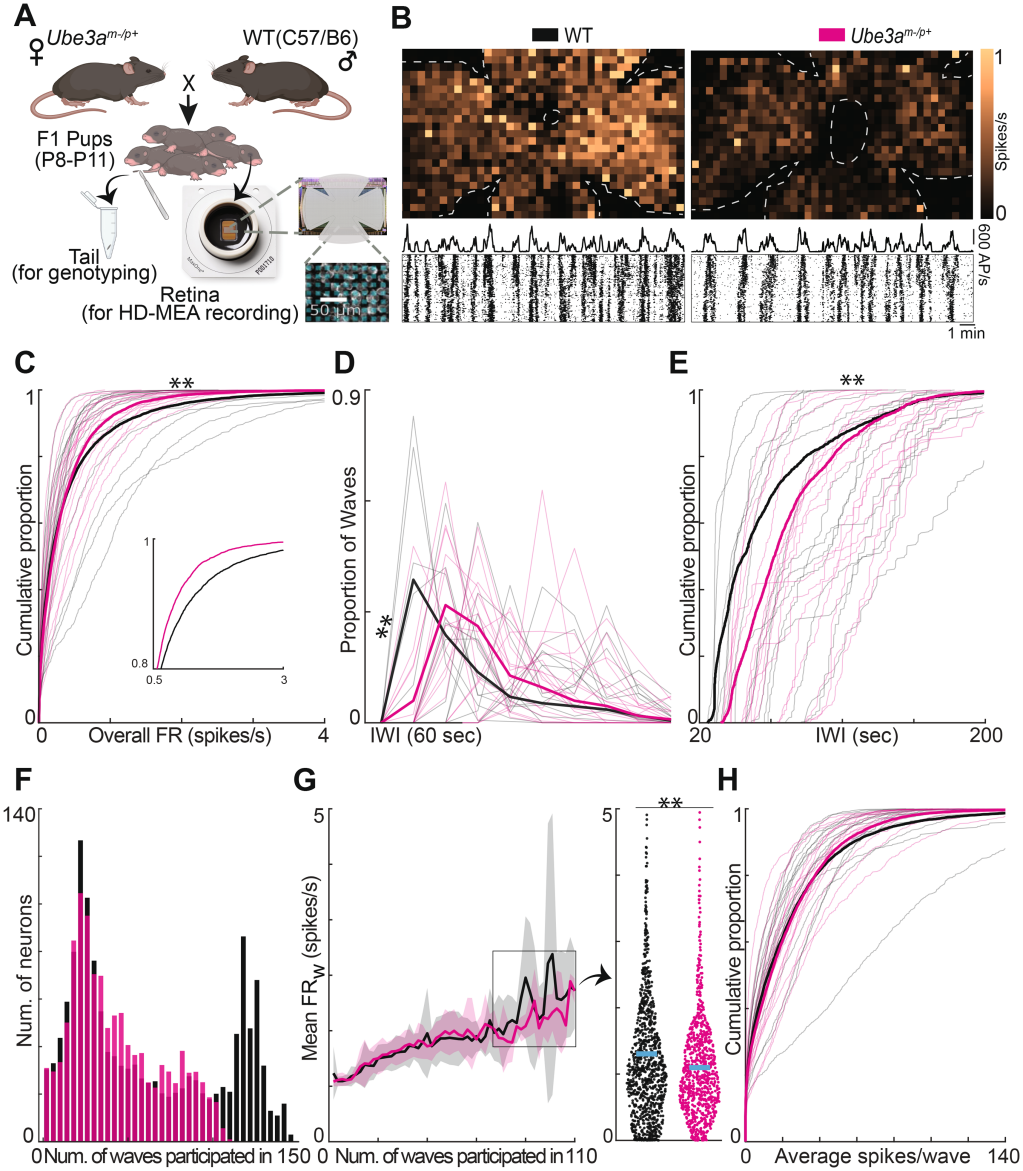
HD-MEA recordings reveal reduced firing rates and altered wave dynamics in AS RGCs during early postnatal development. **(A)** Schematic of experimental workflow. Retinas from F1 pups (P8–P11), generated by crossing AS females (*Ube3a^m−/p+^*) and WT (C57BL/6) males, were used for high-density microelectrode array (HD-MEA) recordings. **(B)** Representative activity recorded from WT and AS retinas. Heatmaps illustrate the mean RGC firing rate (spikes/s) (with normalized color intensity) across the array, averaged over the entire recording duration (top panel). Population instantaneous firing rate, calculated as the total spike count across all detected neurons per time bin (middle panel), where periodic peaks correspond to retinal wave events. Raster plots represent spike times of individual RGCs, bouts of coordinated activity characteristic of retinal waves (lower panel). **(C)** Cumulative distribution plots of overall mean firing rates between genotypes, inset shows a magnified view of the upper tail of the distribution (firing rates > 1.0 spikes/s), highlighting that the difference between genotypes is most pronounced at higher firing rates. **(D)** Inter-wave interval (IWI) distributions of both genotypes, with 60-s mark on the *x*-axis indicates the time bin scale corresponds to 60-s intervals. **(E)** Cumulative distribution plots of IWIs between genotypes. **(F)** Distribution of RGC participation across wave frequencies. **(G)** Relationship between wave participation and mean firing rate during wave (FR_w_). Mean firing rate as a function of waves participated in, with shading representing ± SD across replicates (left panel). Swarm plot of mean firing rates for highly engaged RGCs (≥75 waves) (right panel). Horizontal lines indicate group means. **(H)** Cumulative distribution of average spikes per wave for individual RGCs. (n: WT = 15 retinas, 10,975 RGCs, 2,810 waves, AS = 17 retinas, 11,633 RGCs, 3,243 waves). Data representation: WT in black; AS in magenta. Dark/bold lines represent mean across all retinas for each genotype; faint lines represent individual retinas. (**p < 0.01).

To determine the role of *Ube3a* loss in the development of retinal network activity, we performed HD-MEA recordings from *ex vivo* retinas of P8–P11 mice. We compared littermate control mice (wild-type, WT) with a maternal-deficient *Ube3a* (*Ube3a^m−/p+^*) mouse model of AS (Figures 1A,B; Supplementary Video S1). Representative recordings from WT and AS retinas show the spatial distribution of normalized average firing rates across the retina (Figure 1B, top heatmaps), the instantaneous population firing rate across the entire array (Figure 1B, middle traces), and individual RGC spike times (Figure 1B, bottom raster plots) over the entire 1-h recording period, highlighting the periodic bouts of coordinated activity characteristic of retinal waves.

We first quantified the overall firing rates of individual RGCs. WT RGCs showed a mean firing rate ± SEM of 0.454 ± 0.008 spikes/s, while AS RGCs exhibited a significantly lower mean firing rate of 0.365 ± 0.005 spikes/s (10,975 RGCs from 15 WT mice and 11,633 RGCs from 17 AS mice, *p* < 0.001, Two-sample *t*-test, Figure 1C), representing an approximately 20% reduction in overall firing rate compared to control RGCs.

We next asked whether the reduction in activity in AS mice was due to reduced retinal waves. We used the ‘WaveMiner’ toolbox (Yeager et al., 2025) to segregate individual waves and found a pronounced disruption in the temporal patterning of retinal wave activity. Specifically, we found significantly longer inter-wave intervals (IWI) in AS retina compared to WT [Figures 1D,E; median (inter-quartile ranges—IQR): WT: 48.28 (33.91–77.67) seconds vs. AS 68.19 (51.40–96.38) seconds; Mann–Whitney U: *p <* 0.001], indicating a slower wave frequency and approximately 30% fewer waves in AS retinas.

Besides reduced frequency in retinal waves, could other factors explain the reduced overall activity in AS mice? We quantified the wave engagement of RGCs (how many waves individual RGCs participated in) and observed that RGC engagement counts peaked at approximately 20–30 waves for both genotypes, followed by a progressive decline in the number of RGCs that participated in a large number of waves (Figure 1F). We next tested whether the relationship between an RGC’s level of wave engagement and its firing rate differed between genotypes and made two discoveries. First, in both WT and AS conditions, the mean firing rate showed a positive relationship with wave participation, increasing from approximately 1.0 spikes/s for RGCs participating in ~14 waves to over 2.0 spikes/s for those participating in ≥50 waves. This suggests that RGCs more actively engaged in wave events exhibit higher firing rates. Second, we observed that at high levels of retinal wave participation, the average firing rate was lower in AS retinas than WT retinas, indicating attenuated engagement-dependent firing rate modulation (Figure 1G, right panel; WT: 1.31 ± 0.04 spikes/s, *n* = 1,058 vs. AS: 1.10 ± 0.03, *n* = 844; *p* < 0.001, Two-sample *t*-test). Together, these findings indicate that in the AS model, highly engaged RGCs exhibit reduced firing rates, and fewer RGCs achieve high wave participation levels.

To determine directly whether the reduced firing rates in AS RGCs are accounted for by fewer wave events, or whether RGCs firing rates were less during individual wave events, we calculated the average number of spikes generated per wave for each RGC (Figure 1H). We found no significant difference in the distribution of spikes per wave between genotypes (WT: 22.297 ± 3.634 vs. AS: 20.676 ± 1.783 spikes/wave; *p* = 0.68, *t*-test). This indicates that once recruited into a wave event, AS RGCs fire at rates comparable to WT, and that the overall reduction in firing rate is predominantly driven by the reduced frequency of wave events rather than by a deficit in the firing intensity of RGCs during individual waves.

### Spatiotemporal properties of retinal waves are preserved in AS retinas

**Figure 2:**
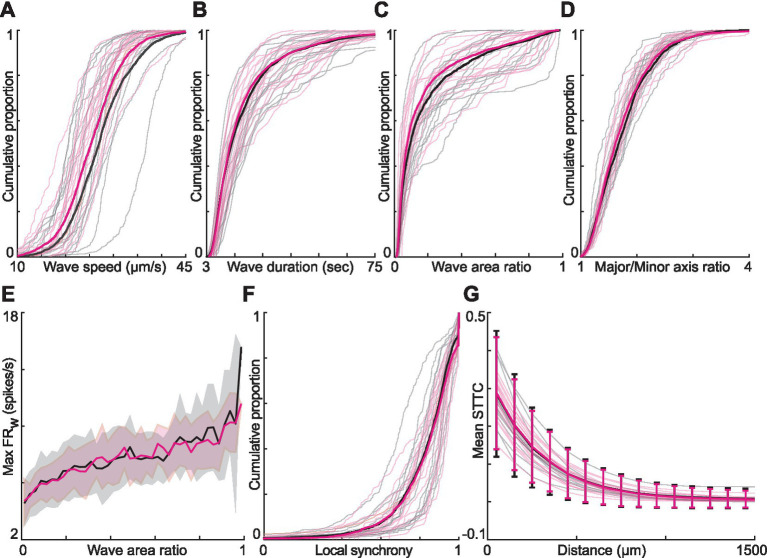
Wave properties are preserved in AS retinas. **(A)** Wave propagation speed distributions between WT and AS. **(B)** Wave duration distributions between WT and AS. **(C)** Wave area ratio distributions between WT and AS. **(D)** Waves’ major/minor axis ratio distributions between WT and AS. **(E)** Relationship between wave area ratio and mean firing rate, with shading representing ± SD across replicates. **(F)** Local synchrony distributions between WT and AS. **(G)** Mean spike-time tiling coefficient (STTC) as a function of pairwise inter-electrode distance between WT and AS. (n: WT = 15 retinas, 10,975 RGCs, 2,810 waves, AS = 17 retinas, 11,633 RGCs, 3,243 waves). Data representation: WT in black; AS in magenta. Dark/bold lines represent mean across all retinas for each genotype; faint lines represent individual retinas.

We next examined whether these differences translated into alterations in the spatial dynamics of spontaneous retinal waves by analyzing the properties of individual waves. We compared five wave parameters between WT and AS retinas: wave propagation speed, duration, wave area ratio, major/minor axis ratio (a measure of wave shape), and local synchrony (the proportion of active RGCs within a wave compared to just outside of the wave) (Figures 2A–F).

Wave propagation speed was not significantly different between genotypes (WT: 26.03 ± 1.11 μm/s, AS: 24.83 ± 0.63 μm/s, *p* = 0.3430, *t*-test) (Figure 2A). Similarly, wave duration was comparable between WT and AS retinas (23.69 ± 1.71 s vs. 21.81 ± 1.04 s, *p* = 0.3419, *t*-test). No significant differences were observed in wave area ratio (WT: 0.219 ± 0.02, AS: 0.197 ± 0.02; *p* = 0.4168, *t*-test), and wave shape (major/minor axis ratio) (WT: 1.739 ± 0.03, AS: 1.746 ± 0.02; *p* = 0.8585, *t*-test) (Figures 2B–D). We also analyzed whether the firing rate of RGCs changed as a function of wave area, and whether this relationship was different between WT and AS mice. Although we made an interesting discovery that RGCs participating in large waves exhibit higher firing rates, this relationship was true for both genotypes (Figure 2E). Next, we analyzed local synchrony (RGCs mean activity ratio in wavefront), which was comparable between WT and AS retinas (WT: 0.827 ± 0.0034, AS: 0.835 ± 0.0031; *p* = 0.102, *t*-test) (Figure 2F). Together, these findings indicate that the intrinsic properties of individual waves remain intact, suggesting that loss of *Ube3a* specifically affects the timing between waves rather than the propagation dynamics of the waves themselves.

We next asked whether *Ube3a* deficiency affects the spatial functional connectivity between RGCs. To analyze this, we quantified pairwise functional connectivity across increasing inter-electrode distance up to 1,500 μm, using the Spike Time Tiling Coefficient (STTC) (Cutts and Eglen, 2014), a correlation metric that measures the degree of temporally correlated firing between RGC pairs beyond what is expected from their individual firing rates. This allowed us to assess whether functional connectivity varied as a function of inter-neuronal distance, and we observed no significant differences between WT and AS retinas (Figure 2G). For the nearest distance bin (0–100 μm), STTC values were comparable between genotypes (WT: 0.2751 ± 0.017; AS: 0.2749 ± 0.013; *p* = 0.9926, *t*-test). Similarly, at all subsequent distance bins up to 1,500 μm, STTC values remained comparable between genotypes.

## Discussion

In this study, we investigated the impact of *Ube3a* deficiency on retinal activity during development, including the firing properties of individual RGCs, the spatiotemporal properties of retinal waves, and the distance-based pairwise correlated activity among RGCs. Our findings reveal that *Ube3a* loss leads to a significant reduction in overall RGC firing rates and number of retinal waves without alterations in wave propagation properties and pairwise spike time correlations between RGCs.

### Reduced retinal activity

The most striking finding is the significant ~20% reduction in the mean firing rate AS RGCs over the course of a 1-h recording, driven largely by a reduction in retinal wave frequency (one-third fewer waves). Extrapolated over the days that retinal waves occupy during visual system development, our result represents a substantial reduction in patterned retinal output to downstream visual centers. The observation that wave frequency is reduced while propagation properties remain intact suggests that Ube3a loss specifically impairs wave initiation rather than the intercellular signaling mechanisms that propagate activity across the retina.

A critical question arising from the reduced overall firing rates and wave frequency in AS retinas is whether this hypoactivity simply reflects a reduction in the frequency of wave events, or do AS RGCs also exhibit reduced firing during individual waves? To determine this, we normalized firing output by wave participation and found no significant difference in spikes per wave between genotypes (Figure 1H), indicating that once recruited into a wave, AS RGCs fire at rates comparable to WT. This finding suggests that the reduced overall firing rate in AS retinas is primarily driven by the reduced frequency of wave events rather than by a deficit in RGC firing rate during individual waves. This further supports the conclusion that the primary deficit in AS retinas lies in wave initiation mechanisms rather than in the ability of RGCs to respond to propagating excitatory drive in the developing retina.

Multiple molecular mechanisms could account for this hypoactivity. First, UBE3A is strongly expressed in both RGCs and amacrine cells in the mouse retina (Li et al., 2024). UBE3A regulates degradation of Activity-regulated cytoskeleton-associated (Arc) protein, and its loss leads to excessive Arc accumulation, reduced surface AMPA receptor expression, and weakened excitatory synaptic transmission in hippocampal neurons (Greer et al., 2010). However, because our recordings at P8–P11 likely also capture Stage II (cholinergic) waves, AMPA receptor-dependent mechanisms may not fully account for the observed phenotype. Indeed, stochastic depolarization and cholinergic transmission of starburst amacrine cells is critical for the initiation and propagation of Stage II waves (Ford et al., 2012). Whether UBE3A regulates excitability or synaptic protein turnover in starburst amacrine cells or other retinal interneurons remains to be determined.

Another notable finding was the relationship between wave participation and firing rate. In WT retinas, RGC firing rates per wave increased progressively with the number of waves participated in. This positive relationship indicates that WT RGCs with greater wave participation exhibit higher excitability, consistent with a form of engagement-dependent modulation that reinforces firing in neurons well-integrated into network events. Strikingly, AS RGCs displayed consistently lower firing rates across all participation levels, and among the most highly engaged RGCs (≥75 waves), AS neurons showed significantly lower firing rates compared to WT, suggesting that *Ube3a* loss limits firing capacity even when RGCs participate in wave events.

### Disrupted temporal but preserved spatial properties of waves

During the developmental window examined here (P8–P11), retinal waves are a mix of Stage II, driven by cholinergic transmission from starburst amacrine cells (SACs) that propagate through recurrent excitatory connections among SACs (Feller et al., 1996; Zheng et al., 2006; Ford et al., 2012) and Stage III, driven by glutamatergic transmission from bipolar cells (Li et al., 2024) which are both spontaneously and light-triggered (Tiriac et al., 2018). AS retinas exhibited significant longer IWIs, indicating a fundamental shift in the operating point of the retinal network and suggesting a possible disruption in the network’s recurrent excitatory mechanisms. Furthermore, despite the disruption in wave timing, we found that the intrinsic properties of individual waves, including speed, duration, area ratio, shape, and local synchrony, remained intact in AS retinas. Intact wave speed and duration suggest that the recurring connections between starburst amacrine cells are mostly intact (Ford et al., 2012; Lansdell et al., 2014). The preserved wave area and shape further suggest uniform retinal network topography (Stafford et al., 2009; Blankenship and Feller, 2010; Maccione et al., 2014). The preserved wave propagation properties in AS suggest that once a wave is initiated, the downstream neuron-to-neuron signaling is likely intact in AS mice.

Despite reductions in firing rates and disrupted wave timing, we did not find any significant differences in the pairwise correlation of RGCs in AS compared to WT (STTC analysis). Combined with the local synchrony results, which are a measure of how much of the activity is within the wavefront versus local retinal space, these results continue to indicate two things related to the spatial nature of retinal waves: the wave-like property of retinal waves is intact in AS mice and the overall amount of correlated activity stems from the synchronized activity of nearby neurons that are recruited by waves. Together, these results reflect likely intact retinal circuitry and anatomy despite UBE3A loss.

### Implications for visual system development and AS pathophysiology

Visual system abnormalities, including strabismus and nystagmus, are common in individuals with AS but are often overshadowed by more prominent neurological features. Our findings suggest that these visual deficits may have a developmental origin rooted in the retina itself. Retinal waves are the primary source of patterned activity that drives the activity-dependent refinement of retinofugal projections (Stafford et al., 2009). The observed reduction in retinal waves indicates a reduced instructional signal transmitted from the *Ube3a*-deficient retina to the developing brain during the critical early developmental window, which could explain *Ube3a*’s role in contributing to the visual processing impairments, poor eye contact, and attention deficits commonly observed in individuals with AS. Such precisely known perturbations lead to defective wiring in the superior colliculus (SC) (Mrsic-Flogel et al., 2005), dorsal lateral geniculate nucleus (dLGN), and visual cortex (Burbridge et al., 2024).

Our study has several key limitations. First, although retinal waves undergo significant developmental changes across the first two postnatal weeks, transitioning from cholinergic (Stage II) to glutamatergic (Stage III) mechanisms (Feller, 1999; Blankenship and Feller, 2010), our study is focused on analyzing P8–P11 developmental window because it captures the overlap of both mechanisms, maximizing our ability to detect deficits. However, whether waves are also affected during the first postnatal week or embryonically, and when the hypoactivity first emerges, remains to be determined. Furthermore, whether the deficits we observe persist, worsen, or recover at later stages (e.g., after eye-opening) remains unknown. Additionally, SACs are the primary cell type responsible for driving Stage II waves (Feller et al., 1996; Ford et al., 2012). Whether SACs are the cell type most affected, or whether connectivity between SACs and RGCs is disrupted given UBE3A’s known synaptic role, remains to be determined. Addressing these questions will require a comprehensive longitudinal analysis spanning the full developmental trajectory of retinal waves, which is a critical direction for future work. Such studies will also help determine whether the primary defect lies in cholinergic mechanisms (e.g., starburst amacrine cell excitability or synaptic transmission), glutamatergic mechanisms (e.g., bipolar cell function), or both. The broad expression profile of Ube3a has been established in adult mouse retinal cells, including RGCs (Rheaume et al., 2018) and amacrine cell subtypes (Yan et al., 2020; Choi et al., 2023); however, the lack of cell-type-resolved data on *Ube3a* expression and imprinting status in the developing mouse retina at P8–P11 limits our mechanistic interpretation. Furthermore, the highly engaged RGCs in our analysis may represent a subset of subtypes, and *Ube3a* deficiency could have subtype-specific effects that are masked in population averages. Studies using genetic labeling and Cre-dependent or optogenetic RGC subtype activation could address this question. Future work could employ visual stimulation or pharmacological modulation during this developmental window to determine which neurotransmitter systems are primarily affected in AS retinas, and whether the phenotype can be rescued.

## Conclusion

In this study, we demonstrate that *Ube3a* deficiency in a mouse model of AS leads to reduced RGC firing rates, prolonged IWIs, and impaired engagement-dependent firing rate modulation, while preserving wave propagation properties and spatial functional connectivity. Despite reduced firing rates, spatial functional connectivity remained intact, indicating that Ube3a loss affects synaptic strength rather than architecture of functional connectivity. The preserved connectivity provides a foundation for therapeutic strategies aimed at normalizing excitability in preclinical AS models. Future work examining the cellular mechanisms underlying reduced excitability and the developmental trajectory of these deficits will further clarify how *Ube3a* regulates network activity and identify optimal windows for therapeutic intervention.

## Methods

### Animals

*Ube3a^tm1Alb/J^* mice (AS model) with maternal allele transmission of the mutation (maternal allele knocked out) were purchased from The Jackson Laboratory (stock No. 016590, Bar Harbor, ME). A breeding colony was established, and the pups were genotyped by PCR-based genotyping using The Jackson Laboratory protocol. Age-matched wild-type littermates served as controls. In this study, all the experiments were conducted on P8-P11 mice of both sexes and were housed in a 12-h day-night cycle with access to food and water. All animal procedures were approved by the Vanderbilt Animal Care and Use Program and conformed to the NIH Guide for the Care and Use of Laboratory Animals, the Public Health Service Policy, and the SFN Policy on the Use of Animals in Neuroscience Research.

### Retinal preparation

Following euthanasia by isoflurane inhalation overdose (Piramal Pharma Limited, India), eyes were enucleated. Retinas were dissected in a Petri dish containing ice-cold oxygenated aCSF (in mM, 119 NaCl, 2.5 KCl, 1.3 MgCl_2_, 1 K_2_HPO_4_, 26.2 NaHCO_3_, 11 D-glucose, and 2.5 CaCl_2_) under a dissection microscope (Zeiss, Stemi 508, White Plains, NY) and placed RGC-side-down on a single-well MaxOne HD-MEA chip (MaxWell Biosystems, Switzerland) with the ventral retina oriented downward (Gupta et al., 2025). The HD-MEA chip was quickly transferred onto the MaxOne Tissue Holder (MaxWell Biosystems, Switzerland), and then a replaceable tissue holder insert was used to gently press the retina against the electrodes and secure it. A perfusion system (Ismatec, ISM4208) was used to perfuse oxygenated aCSF at a constant flow rate (2 mL/min). Using an inline heater (Multichannel System, TC02) in the perfusion system, the aCSF temperature was maintained at 32–34 °C to maintain physiological conditions.

### High-density microelectrode array (HD-MEA) recordings

Spontaneous retinal activity was recorded using the MaxOne HD-MEA chip (MaxWell Biosystems, Switzerland), a complementary metal-oxide-semiconductor (CMOS)-based HD-MEA platform. The MaxOne chip contains ~26,000 platinum electrodes (electrode size: 11.5 × 11.5 μm^2^; center-to-center distance: 17.5 μm) spanning a sensing area of approximately 3.85 × 2.10 mm^2^, of which ~1,020 electrodes can be recorded simultaneously through on-chip routing. The high electrode density provides single-cell spatial resolution, with multiple electrodes typically capturing signals from individual RGCs. Following placement of the retina RGC-side-down on the chip surface, the tissue was gently secured against the electrodes using a replaceable tissue holder insert. A MaxOne Hub (MaxWell Biosystems, Switzerland) recording unit was connected to the MaxOne Tissue Holder. Following a 1-h acclimatization period, ensuring tissue stability, spontaneous retinal activity of RGCs detected by the electrodes on the MaxOne chip was recorded for 1-h at 20 kHz sampling rate, and spikes were detected using a standard threshold method. Firing rates (spikes/s) were calculated for each neuron and averaged across all neurons per retina. For visualization of network activity over time, instantaneous firing rate traces were computed by binning spike times across all RGCs in 1-s bins and calculating the total spike count per bin, yielding a time series of population firing rate in spikes/s. This approach captures the temporal dynamics of network activity, including the periodic bursts characteristic of retinal waves. Retinal waves were identified using the ‘WaveMiner’ toolbox developed by our lab to study the spatiotemporal properties of the retinal waves (Yeager et al., 2025). The following wave parameters were quantified: wave speed: the propagation velocity (μm/s) across the retina; wave duration: temporal length of the wave event (s); wave area ratio (the fraction of the recording area covered by a wave); wave shape: the major-to-minor axis ratio; inter-wave interval (per pixel): time between the start of consecutive waves cross over a pixel (s).

### Statistical analysis

All statistical analyses were performed using custom MATLAB (R2024b) scripts. Data are presented as mean ± standard error of mean (SEM), unless otherwise stated. For wave-modulated firing, recording periods were classified as “during wave” or “tonic firing.” Pairwise functional connectivity was assessed using the Spike Time Tiling Coefficient (STTC) (Cutts and Eglen, 2014), a correlation metric that is robust to quantify the temporal correlation between spike trains of neuron pairs. STTC measures the degree to which spikes from two neurons occur within a defined temporal window of each other, with values ranging from −1 to +1, where values near +1 indicate highly correlated firing, values near 0 indicate independence, and negative values indicate anti-correlation. For each retina, STTC was calculated for all unique neuron pairs using a coincidence window of Δt = 50 ms and binned by the physical inter-electrode distance (100 μm bins, from 0 to 1,500 μm). Statistical comparisons between genotypes at each distance bin were conducted using Mann–Whitney U tests. Wave shape was quantified as the major-to-minor axis ratio, computed by performing PCA on the spatial coordinates of wave-occupied pixels and computing the ratio of the square roots of the two largest eigenvalues of the spatial covariance matrix. For all wave parameters, genotype comparisons were performed using the *t*-test. Retina-level averages for wave frequency and vector sum length were compared using the two-sample *t*-test. A *p-value* of < 0.05 was considered statistically significant.

## Data Availability

The raw data supporting the conclusions of this article will be made available by the authors, without undue reservation.

## Supplementary material

SUPPLEMENTARY VIDEO S1 AS retinas exhibit fewer retinal waves. Example data from a WT (Left column) and AS retina (Right column). Panel **(A)** Raw movie of spike rates collected from the HD-MEA. Panel **(B)** Average spike rate over long time-courses using 90 s moving average window. Normalized scale bar in this panel applicable for the panel A as well. Panel **(C)** Segregated waves using the “WaveMiner” toolbox. *x*-axis and *y*-axis in panels **(A–C)**: the spatial axes map the physical location of electrodes across the sensing area of the MEA chip with each pixel corresponding to an individual electrode position. Panel **(D)** Raster plot of action potentials for all neurons (*y* axis) over the course of the recording (*x* axis). Panel **(E)** Sum of action potential calculated from the raster plot.

## References

[ref1] Ackman J. B. Burbridge T. J. Crair M. C. (2012). Retinal waves coordinate patterned activity throughout the developing visual system. Nature 490, 219–225. doi: 10.1038/nature11529, 23060192 PMC3962269

[ref2] Biswas S. Shahriar S. Bachay G. Arvanitis P. Jamoul D. Brunken W. J. . (2024). Glutamatergic neuronal activity regulates angiogenesis and blood-retinal barrier maturation via Norrin/beta-catenin signaling. Neuron 112, 1978–1996.e6. doi: 10.1016/j.neuron.2024.03.011 38599212 PMC11189759

[ref3] Blankenship A. G. Feller M. B. (2010). Mechanisms underlying spontaneous patterned activity in developing neural circuits. Nat. Rev. Neurosci. 11, 18–29. doi: 10.1038/nrn2759, 19953103 PMC2902252

[ref4] Burbridge T. J. Ratliff J. M. Dwivedi D. Vrudhula U. Alvarado-Huerta F. Sjulson L. . (2024). Disruption of cholinergic retinal waves alters visual cortex development and function. bioRxiv. doi: 10.1101/2024.04.05.588143, 38644996 PMC11030223

[ref5] Chandrasekaran A. R. Plas D. T. Gonzalez E. Crair M. C. (2005). Evidence for an instructive role of retinal activity in retinotopic map refinement in the superior colliculus of the mouse. J. Neurosci. 25, 6929–6938. doi: 10.1523/JNEUROSCI.1470-05.2005, 16033903 PMC6725341

[ref6] Choi J. Li J. Ferdous S. Liang Q. Moffitt J. R. Chen R. (2023). Spatial organization of the mouse retina at single cell resolution by MERFISH. Nat. Commun. 14:4929. doi: 10.1038/s41467-023-40674-3, 37582959 PMC10427710

[ref7] Cutts C. S. Eglen S. J. (2014). Detecting pairwise correlations in spike trains: an objective comparison of methods and application to the study of retinal waves. J. Neurosci. 34, 14288–14303. doi: 10.1523/JNEUROSCI.2767-14.2014, 25339742 PMC4205553

[ref8] Feller M. B. (1999). Spontaneous correlated activity in developing neural circuits. Neuron 22, 653–656. doi: 10.1016/S0896-6273(00)80724-2, 10230785

[ref9] Feller M. B. Wellis D. P. Stellwagen D. Werblin F. S. Shatz C. J. (1996). Requirement for cholinergic synaptic transmission in the propagation of spontaneous retinal waves. Science 272, 1182–1187. doi: 10.1126/science.272.5265.1182, 8638165

[ref10] Ford K. J. Felix A. L. Feller M. B. (2012). Cellular mechanisms underlying spatiotemporal features of cholinergic retinal waves. J. Neurosci. 32, 850–863. doi: 10.1523/JNEUROSCI.5309-12.2012, 22262883 PMC3311224

[ref11] Galli J. Loi E. Strobio C. Micheletti S. Martelli P. Merabet L. B. . (2023). Neurovisual profile in children affected by Angelman syndrome. Brain Dev. 45, 117–125. doi: 10.1016/j.braindev.2022.10.003, 36344336

[ref12] Ge X. Zhang K. Gribizis A. Hamodi A. S. Sabino A. M. Crair M. C. (2021). Retinal waves prime visual motion detection by simulating future optic flow. Science 373:eabd0830. doi: 10.1126/science.abd0830, 34437090 PMC8841103

[ref13] Greer P. L. Hanayama R. Bloodgood B. L. Mardinly A. R. Lipton D. M. Flavell S. W. . (2010). The Angelman syndrome protein Ube3A regulates synapse development by ubiquitinating arc. Cell 140, 704–716. doi: 10.1016/j.cell.2010.01.026, 20211139 PMC2843143

[ref14] Gupta R. K. Clark N. Yeager K. Fisch A. Tiriac A. (2025). High-density multielectrode array recordings of retinal waves using an electrophysiology platform. J. Vis. Exp. doi: 10.3791/68493, 40658718 PMC12710770

[ref15] Huberman A. D. Feller M. B. Chapman B. (2008). Mechanisms underlying development of visual maps and receptive fields. Annu. Rev. Neurosci. 31, 479–509. doi: 10.1146/annurev.neuro.31.060407.125533, 18558864 PMC2655105

[ref16] Jiang Y. H. Armstrong D. Albrecht U. Atkins C. M. Noebels J. L. Eichele G. . (1998). Mutation of the Angelman ubiquitin ligase in mice causes increased cytoplasmic p53 and deficits of contextual learning and long-term potentiation. Neuron 21, 799–811. doi: 10.1016/s0896-6273(00)80596-6 9808466

[ref17] Jiang Y. H. Pan Y. Zhu L. Landa L. Yoo J. Spencer C. . (2010). Altered ultrasonic vocalization and impaired learning and memory in Angelman syndrome mouse model with a large maternal deletion from Ube3a to Gabrb3. PLoS One 5:e12278. doi: 10.1371/journal.pone.0012278, 20808828 PMC2924885

[ref18] Lansdell B. Ford K. Kutz J. N. (2014). A reaction-diffusion model of cholinergic retinal waves. PLoS Comput. Biol. 10:e1003953. doi: 10.1371/journal.pcbi.1003953, 25474327 PMC4256014

[ref19] Li J. Choi J. Cheng X. Ma J. Pema S. Sanes J. R. . (2024). Comprehensive single-cell atlas of the mouse retina. iScience 27:109916. doi: 10.1016/j.isci.2024.109916, 38812536 PMC11134544

[ref20] Maccione A. Hennig M. H. Gandolfo M. Muthmann O. Van Coppenhagen J. Eglen S. J. . (2014). Following the ontogeny of retinal waves: pan-retinal recordings of population dynamics in the neonatal mouse. J. Physiol. 592, 1545–1563. doi: 10.1113/jphysiol.2013.262840, 24366261 PMC3979611

[ref21] Margolis S. S. Sell G. L. Zbinden M. A. Bird L. M. (2015). Angelman Syndrome. Neurotherapeutics 12, 641–650. doi: 10.1007/s13311-015-0361-y, 26040994 PMC4489961

[ref22] Matsumoto N. Barson D. Liang L. Crair M. C. (2024). Hebbian instruction of axonal connectivity by endogenous correlated spontaneous activity. Science 385:eadh7814. doi: 10.1126/science.adh7814, 39146415 PMC12148345

[ref23] Mclaughlin T. Torborg C. L. Feller M. B. O'leary D. D. (2003). Retinotopic map refinement requires spontaneous retinal waves during a brief critical period of development. Neuron 40, 1147–1160. doi: 10.1016/s0896-6273(03)00790-6, 14687549

[ref24] Michieletto P. Bonanni P. Pensiero S. (2011). Ophthalmic findings in Angelman syndrome. J. AAPOS 15, 158–161. doi: 10.1016/j.jaapos.2010.12.013, 21596294

[ref25] Mrsic-Flogel T. D. Hofer S. B. Creutzfeldt C. Cloez-Tayarani I. Changeux J. P. Bonhoeffer T. . (2005). Altered map of visual space in the superior colliculus of mice lacking early retinal waves. J. Neurosci. 25, 6921–6928. doi: 10.1523/JNEUROSCI.1555-05.2005, 16033902 PMC6725344

[ref26] Ozarkar S. S. Patel R. K. Vulli T. Smith A. L. Styner M. A. Hsu L. M. . (2024). Comparative profiling of white matter development in the human and mouse brain reveals volumetric deficits and delayed myelination in Angelman syndrome. Mol. Autism. 15:54. doi: 10.1186/s13229-024-00636-y 39726042 PMC11670556

[ref27] Pelc K. Cheron G. Dan B. (2008). Behavior and neuropsychiatric manifestations in Angelman syndrome. Neuropsychiatr. Dis. Treat. 4, 577–584. doi: 10.2147/ndt.s2749, 18830393 PMC2526368

[ref28] Rheaume B. A. Jereen A. Bolisetty M. Sajid M. S. Yang Y. Renna K. . (2018). Single cell transcriptome profiling of retinal ganglion cells identifies cellular subtypes. Nat. Commun. 9:2759. doi: 10.1038/s41467-018-05134-3, 30018341 PMC6050223

[ref29] Shi S. Q. Bichell T. J. Ihrie R. A. Johnson C. H. (2015). Ube3a imprinting impairs circadian robustness in Angelman syndrome models. Curr. Biol. 25, 537–545. doi: 10.1016/j.cub.2014.12.047, 25660546 PMC4348236

[ref30] Sidorov M. S. Judson M. C. Kim H. Rougie M. Ferrer A. I. Nikolova V. D. . (2018). Enhanced operant extinction and prefrontal excitability in a mouse model of Angelman syndrome. J. Neurosci. 38, 2671–2682. doi: 10.1523/JNEUROSCI.2828-17.2018, 29431654 PMC5852653

[ref31] Stafford B. K. Sher A. Litke A. M. Feldheim D. A. (2009). Spatial-temporal patterns of retinal waves underlying activity-dependent refinement of retinofugal projections. Neuron 64, 200–212. doi: 10.1016/j.neuron.2009.09.021, 19874788 PMC2771121

[ref32] Stellwagen D. Shatz C. J. (2002). An instructive role for retinal waves in the development of retinogeniculate connectivity. Neuron 33, 357–367. doi: 10.1016/S0896-6273(02)00577-9, 11832224

[ref33] Thompson A. Gribizis A. Chen C. Crair M. C. (2017). Activity-dependent development of visual receptive fields. Curr. Opin. Neurobiol. 42, 136–143. doi: 10.1016/j.conb.2016.12.007, 28088066 PMC5375035

[ref34] Tiriac A. Smith B. E. Feller M. B. (2018). Light prior to eye opening promotes retinal waves and eye-specific segregation. Neuron 100, 1059–1065.e4. doi: 10.1016/j.neuron.2018.10.011, 30392793 PMC6283698

[ref35] Townsend L. B. Jones K. A. Dorsett C. R. Philpot B. D. Smith S. L. (2020). Deficits in higher visual area representations in a mouse model of Angelman syndrome. J. Neurodev. Disord. 12:28. doi: 10.1186/s11689-020-09329-y, 33076843 PMC7574469

[ref36] Vihma H. Li K. Welton-Arndt A. Smith A. L. Bettadapur K. R. Gilmore R. B. . (2024). Ube3a unsilencer for the potential treatment of Angelman syndrome. Nat. Commun. 15:5558. doi: 10.1038/s41467-024-49788-8, 38977672 PMC11231141

[ref37] Voufo C. Chen A. Q. Smith B. E. Yan R. Feller M. B. Tiriac A. (2023). Circuit mechanisms underlying embryonic retinal waves. eLife 12:e81983. doi: 10.7554/eLife.81983, 36790167 PMC9988258

[ref38] Wallace M. L. Burette A. C. Weinberg R. J. Philpot B. D. (2012). Maternal loss of Ube3a produces an excitatory/inhibitory imbalance through neuron type-specific synaptic defects. Neuron 74, 793–800. doi: 10.1016/j.neuron.2012.03.036, 22681684 PMC3372864

[ref39] Weiner G. A. Shah S. H. Angelopoulos C. M. Bartakova A. B. Pulido R. S. Murphy A. . (2019). Cholinergic neural activity directs retinal layer-specific angiogenesis and blood retinal barrier formation. Nat. Commun. 10:2477. doi: 10.1038/s41467-019-10219-8, 31171770 PMC6554348

[ref40] Williams C. A. Zori R. T. Stone J. W. Gray B. A. Cantu E. S. Ostrer H. (1990). Maternal origin of 15q11-13 deletions in Angelman syndrome suggests a role for genomic imprinting. Am. J. Med. Genet. 35, 350–353. doi: 10.1002/ajmg.1320350308, 2309781

[ref41] Yan W. Laboulaye M. A. Tran N. M. Whitney I. E. Benhar I. Sanes J. R. (2020). Mouse retinal cell atlas: molecular identification of over sixty Amacrine cell types. J. Neurosci. 40, 5177–5195. doi: 10.1523/JNEUROSCI.0471-20.2020, 32457074 PMC7329304

[ref42] Yashiro K. Riday T. T. Condon K. H. Roberts A. C. Bernardo D. R. Prakash R. . (2009). Ube3a is required for experience-dependent maturation of the neocortex. Nat. Neurosci. 12, 777–783. doi: 10.1038/nn.2327, 19430469 PMC2741303

[ref43] Yeager K. M. Gupta R. K. Gauthier E. A. Fisch A. J. Clark N. A. Orsi F. S. . (2025). A toolbox for navigating and analyzing the spatiotemporal properties of retinal waves. bioRxiv. doi: 10.1101/2025.10.23.684204

[ref44] Zheng J. Lee S. Zhou Z. J. (2006). A transient network of intrinsically bursting starburst cells underlies the generation of retinal waves. Nat. Neurosci. 9, 363–371. doi: 10.1038/nn1644, 16462736

